# A new semisynthetic cardenolide analog 3β-[2-(1-amantadine)- 1-on-ethylamine]-digitoxigenin (AMANTADIG) affects G2/M cell cycle arrest and miRNA expression profiles and enhances proapoptotic survivin-2B expression in renal cell carcinoma cell lines

**DOI:** 10.18632/oncotarget.14644

**Published:** 2017-01-14

**Authors:** Elke Nolte, Sven Wach, Izabella Thais Silva, Sabine Lukat, Arif B. Ekici, Jennifer Munkert, Frieder Müller-Uri, Wolfgang Kreis, Cláudia Maria Oliveira Simões, Julio Vera, Bernd Wullich, Helge Taubert, Xin Lai

**Affiliations:** ^1^ Department of Urology, University Hospital Erlangen, Erlangen, Germany; ^2^ Department of Pharmaceutical Sciences, Universidade Federal de Santa Catarina, Florianópolis, Brazil; ^3^ Institute of Human Genetics, Friedrich-Alexander-Universität Erlangen-Nürnberg, Erlangen, Germany; ^4^ Department of Biology, Chair of Pharmaceutical Biology, Friedrich-Alexander-University Erlangen-Nürnberg, Erlangen, Germany; ^5^ Laboratory of Systems Tumor Immunology, Department of Dermatology, University Hospital Erlangen, Friedrich-Alexander-University Erlangen-Nürnberg, Erlangen, Germany; ^6^ Department of Pharmacy, Federal University of Minas Gerais, Belo Horizonte, Brazil

**Keywords:** 3β, -[2-(1-amantadine)-1-on-ethylamine]-digitoxigenin, cardiac glycoside analog, human renal cell carcinoma cells, miRNA, cell cycle

## Abstract

Cardiac glycosides are well known in the treatment of cardiovascular diseases; however, their application as treatment option for cancer patients is under discussion. We showed that the cardiac glycoside digitoxin and its analog AMANTADIG can inhibit the growth of renal cell carcinoma (RCC) cell lines and increase G2/M cell cycle arrest. To identify the signaling pathways and molecular basis of this G2/M arrest, microRNAs were profiled using microRNA arrays. Cardiac glycoside treatment significantly deregulated two microRNAs, miR-2278 and miR-670-5p. Pathway enrichment analysis showed that all cardiac glycoside treatments affected the MAPK and the axon guidance pathway. Within these pathways, three genes, MAPK1, NRAS and RAC2, were identified as *in silico* targets of the deregulated miRNAs. MAPK1 and NRAS are known regulators of G2/M cell cycle arrest. AMANTADIG treatment enhanced the expression of phosphorylated MAPK1 in 786-O cells. Secondly, we studied the expression of survivin known to be affected by cardiac glycosides and to regulate the G2/M cell phase. AMANTADIG treatment upregulated the expression of the pro-apoptotic *survivin-2B* variant in Caki-1 and 786-O cells. Moreover, treatment with AMANTADIG resulted in significantly lower survivin protein expression compared to 786-O control cells. Summarizing, treatment with all cardiac glycosides induced G2/M cell cycle arrest and downregulated the miR-2278 and miR-670-5p in microarray analysis. All cardiac glycosides affected the MAPK-pathway and survivin expression, both associated with the G2/M phase. Because cells in the G2/M phase are radio- and chemotherapy sensitive, cardiac glycosides like AMANTADIG could potentially improve the efficacy of radio- and/or chemotherapy in RCCs.

## INTRODUCTION

Renal Cell Carcinoma (RCC) is the ninth most common cancer worldwide, with approximately 338,000 new cases diagnosed in 2012 [1]. Metastatic renal cell carcinoma retains an especially poor prognosis despite surgical cytoreduction, VEGF-tyrosine kinase inhibitors, mTOR inhibitors and initially promising immunotherapy results [reviewed in 2, 3], which implies an urgent need for additional therapies.

Cardiac glycosides have garnered attention as a potential cancer treatment option [4, 5]. They exhibit anti-proliferative and apoptotic characteristics in several cancer cell lines, including renal cell carcinoma cell lines, but they affect normal cell lines to a much lesser extent [6–10]. We recently showed that a new semisynthetic cardiac glycoside analog, 3β-[2-(1-amantadine)-1-on-ethylamine]-digitoxigenin (AMANTADIG), inhibits the growth of leukemia, prostate cancer and renal cell carcinoma cell lines [11]. Although several reports have indicated that cardiac glycosides may function by affecting Na^+^/K^+^-ATPase, especially its α subunits [4, 5], the molecular basis of their function remains incompletely understood.

In this study, we investigated the functional effects of AMANTADIG on the cell cycle and its molecular effect on gene regulation based on microRNA (miRNA) deregulation. MiRNAs are small, ubiquitous non-coding RNAs that are 17–25 nucleotides in length and play an important regulatory role in many normal and pathophysiological cellular processes, such as cell proliferation, differentiation, the induction of apoptosis, tumorigenesis and tumor progression [12, 13]. MiRNA expression profiles can be used to classify human cancers and delineate their function as tumor suppressors or oncogenes [14, 15], and the role of miRNAs as diagnostic, prognostic and predictive markers is well established in renal cell carcinoma [16–20], [reviewed in 21, 22]. The identification and characterization of deregulated miRNAs after cardiac glycoside treatment may give insight into their gene/pathway regulation and consequently help to clarify the molecular basis of their anti-proliferative and anti-apoptotic effects.

## RESULTS

### Inhibition of Na^+^/K^+^-ATPase activity

To investigate effects of AMANTDIG, digitoxin and β-methyldigoxin on Na^+^/K^+^-ATPase activity, all substrates were assayed with Na^+^/K^+^-ATPase α1,2,3 subunit of porcine cortex. The data indicated an increasing affinity towards Na^+^/K^+^-ATPase inhibition from β-methyldigoxin, digitoxin to AMANTADIG, with IC50 values ranging between 16.73 μM and 4.78 μM (Supplementary Figure 1).

### Cell viability

**Figure 1:**
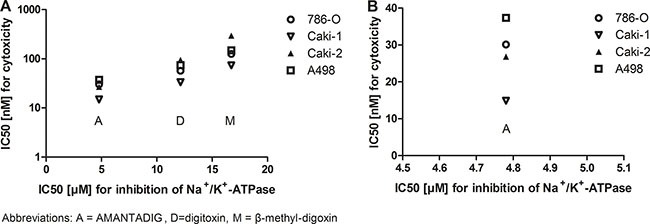
Cytotoxicity effects of cardiac glycosides and inhibition of Na+/K+-ATPase in four RCC cell lines IC50 values for cytotoxicity and IC50 values for Na+/K+ - ATPase inhibition were related to each other after treatment of the four cell lines with the three cardiac glycosides **(A)** or only with AMANTADIG **(B)**.

We studied the effect of cardiac glycoside treatment on the viability of four RCC cell lines, Caki-1, Caki-2, A498 and 786-O and determined the IC50 values. We previously described the effect of treatment with the cardiac glycoside digitoxin and AMANTADIG for 72 h [11]. In this study, we also included the clinically applied cardiac glycoside ß-methyl-digoxin in our analysis. IC50 values were calculated after treatment with the three cardiac glycosides for 24 h, 48 h and 72 h (Table 1). Compared to digitoxin and AMANTADIG, we detected a similar sensitivity of the cell lines to ß-methyl-digoxin. Treatment with each of the three cardiac glycosides for 24 h, 48 h and 72 h identified Caki-1 cells as the most sensitive cell line, and Caki-2 cells as the most resistant cell line (Table 1). Next, we studied if the inhibition activity of the Na^+^/K^+^-ATPase by the cardiac glycosids was correlated to the cytotoxicity in the RCC cell lines. In general, stronger inhibition activity of the Na^+^/K^+^-ATPase was correlated with higher cytotoxicity (48 h) on all RCC cell lines (Figure 1A). In addition, the different cytotoxicity effects (48 h) of AMANTADIG in the RCC cell lines are depicted in Figure 1B.

**Table 1:** Cell viability measured via MTT assay

Treatment/RCC cell lines	digitoxin	24 h		48 h		72 h	
	IC50	95% CI	IC50	95% CI	IC50	95% CI
A498	97.83	78.97 - 121.2	73.94	61.44 - 88.98	40.56	33.60 - 48.96
786-O	100	77.00 - 129.9	57.13	77.00 - 129.9	43.49	38.25 - 49.45
Caki-1	16.11	12.87 - 20.18	33.31	28.59 - 38.81	21.91	18.86 - 25.44
Caki-2	163.7	120.8 - 222.0	94.82	76.87 - 117.0	62.13	37.76 - 102.2
						
ß-methyl-digoxin	24 h		48 h		72 h	
	IC50	95% CI	IC50	95% CI	IC50	95% CI
A498	234.3	193.8 - 283.4	148.5	130.1 - 169.6	100.9	88.53 - 115.1
786-O	311.2	266.6 - 363.3	125.4	112.6 - 139.7	95.98	89.45 - 103.0
Caki-1	121.8	103.7 - 143.0	73.55	65.27 - 82.89	58.88	53.27 - 65.08
Caki-2	180.9	145.8 - 224.4	294.2	230.6 - 375.3	108.3	87.83 - 133.6
						
AMANTADIG	24 h		48 h		72 h	
	IC50	95% CI	IC50	95% CI	IC50	95% CI
A498	87.5	64.69 - 118.4	37.34	27.34 - 50.99	34.09	25.04 - 46.42
786-O	106.6	75.10 - 151.2	30.12	25.32 - 35.82	22.48	17.80 - 28.39
Caki-1	36.49	25.65 - 51.90	14.89	11.13 - 19.91	14.69	11.39 - 18.96
Caki-2	234.5	147.3 - 373.4	26.82	20.57 - 34.96	70.17	47.39 - 103.9

IC50 values for the treatment of renal cell carcinoma cell lines with cardiac glycosides (digitoxin, ß-methyl-digoxin and AMANTADIG). The IC50 values represent the mean and standard deviation from 4 experiments each consisting of three technical replicates (in nM).

### Cell cycle analysis

**Figure 2:**
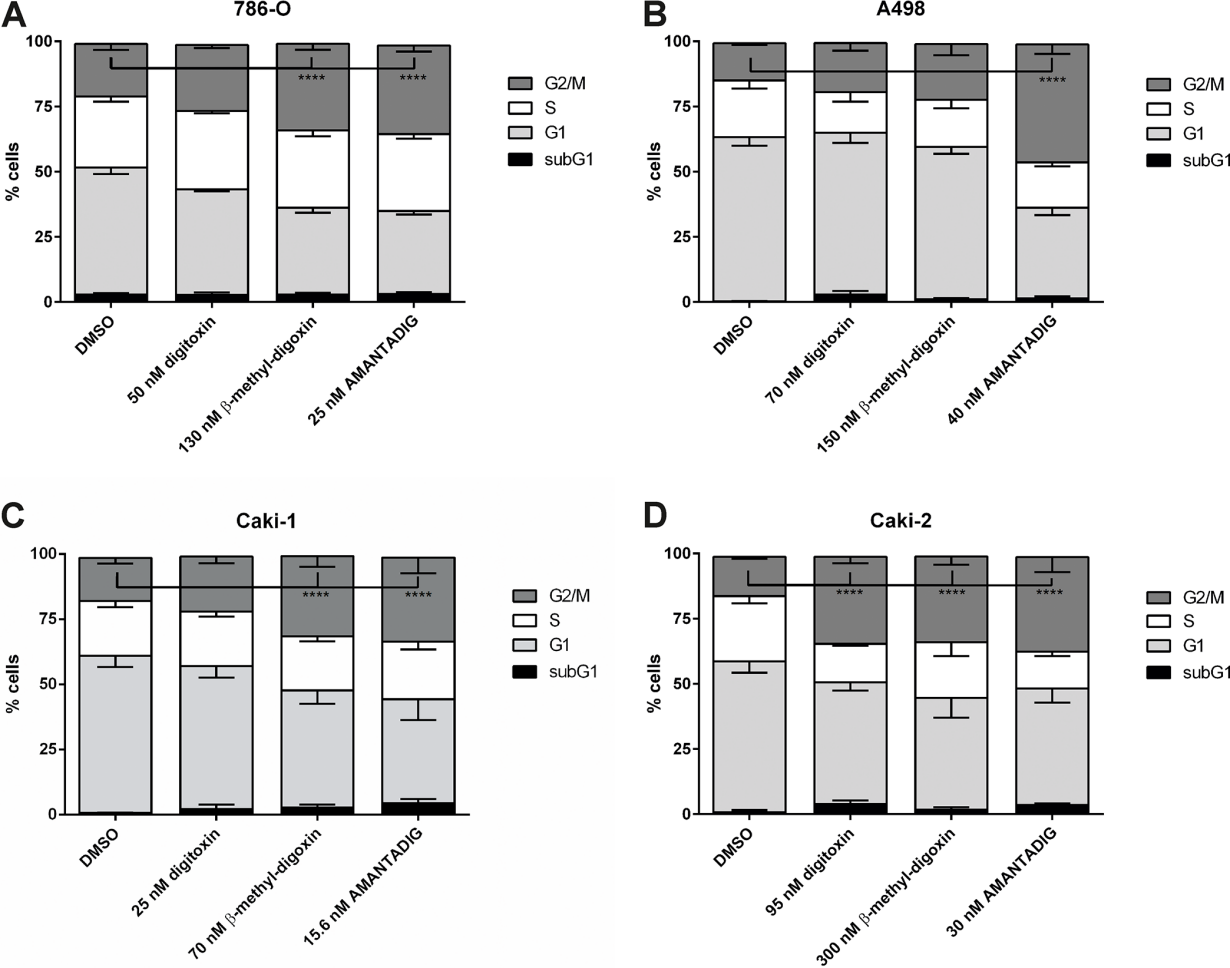
Analysis of the cell cycle after treatment with cardiac glycosides RCC cell lines **(A)** 786-O, **(B)** A498, **(C)** Caki-1 and **(D)** Caki-2 were treated with digitoxin, β-methyl-digoxin and AMANTADIG at their respective IC50 concentrations. Asterisks indicate p < 0.05.

Treatment with cardiac glycosides, including digoxin and digitoxin, have been described to inhibit the cell cycle, especially cell arrest in the G2/M phase of the cell cycle [7, 23]. Therefore, we studied the effect of three cardiac glycosides on the cell cycle after treating four cell lines for 48 h with the IC50 concentrations of each cardiac glycoside (Figure 2). Digitoxin caused significant G2/M arrest only in Caki-2 cells. Treatment with ß-methyl-digoxin resulted in G2/M cell cycle arrest in the three cell lines 786-O, Caki-1 and Caki-2. Most interestingly, treatment with AMANTADIG resulted in significant G2/M arrest in all four renal cell carcinoma cell lines.

### miRNA microarray

**Figure 3:**
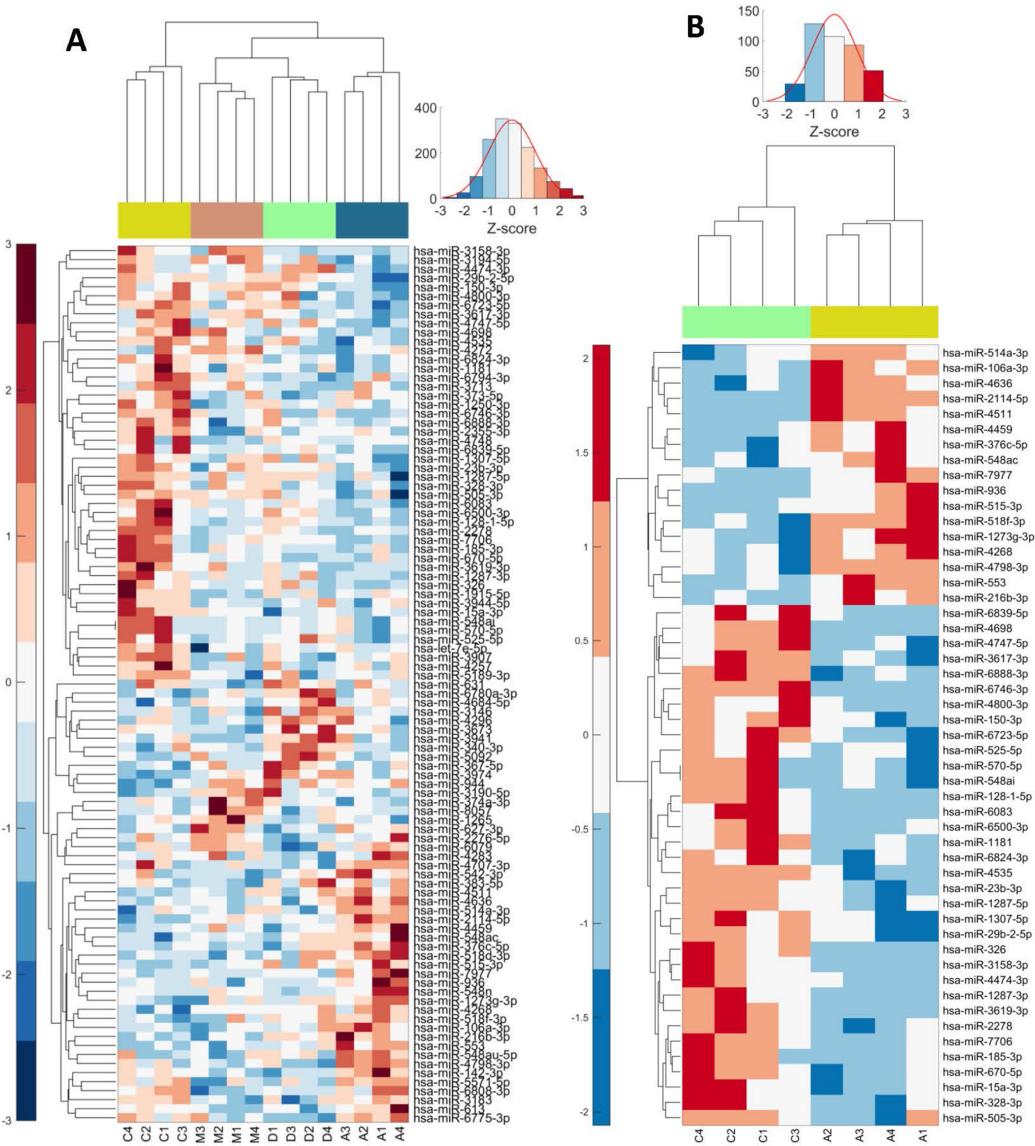
Heat maps: Deregulated miRNAs combined for all compounds or for single AMANTADIG treatment Heat map of deregulated miRNAs after treating the four RCC cell lines with DMSO [C], digitoxin [D], β-methyl-digoxin [M] and AMANTADIG [A]. 1: A498; 2: 786-O; 3: Caki-1; and 4: Caki-2 (A) Heat map of deregulated miRNAs after treating the four RCC cell lines with DMSO [C] or AMANTADIG [A] 1: A498; 2: 786-O; 3: Caki-1; and 4: Caki-2 (B).

**Figure 4:**
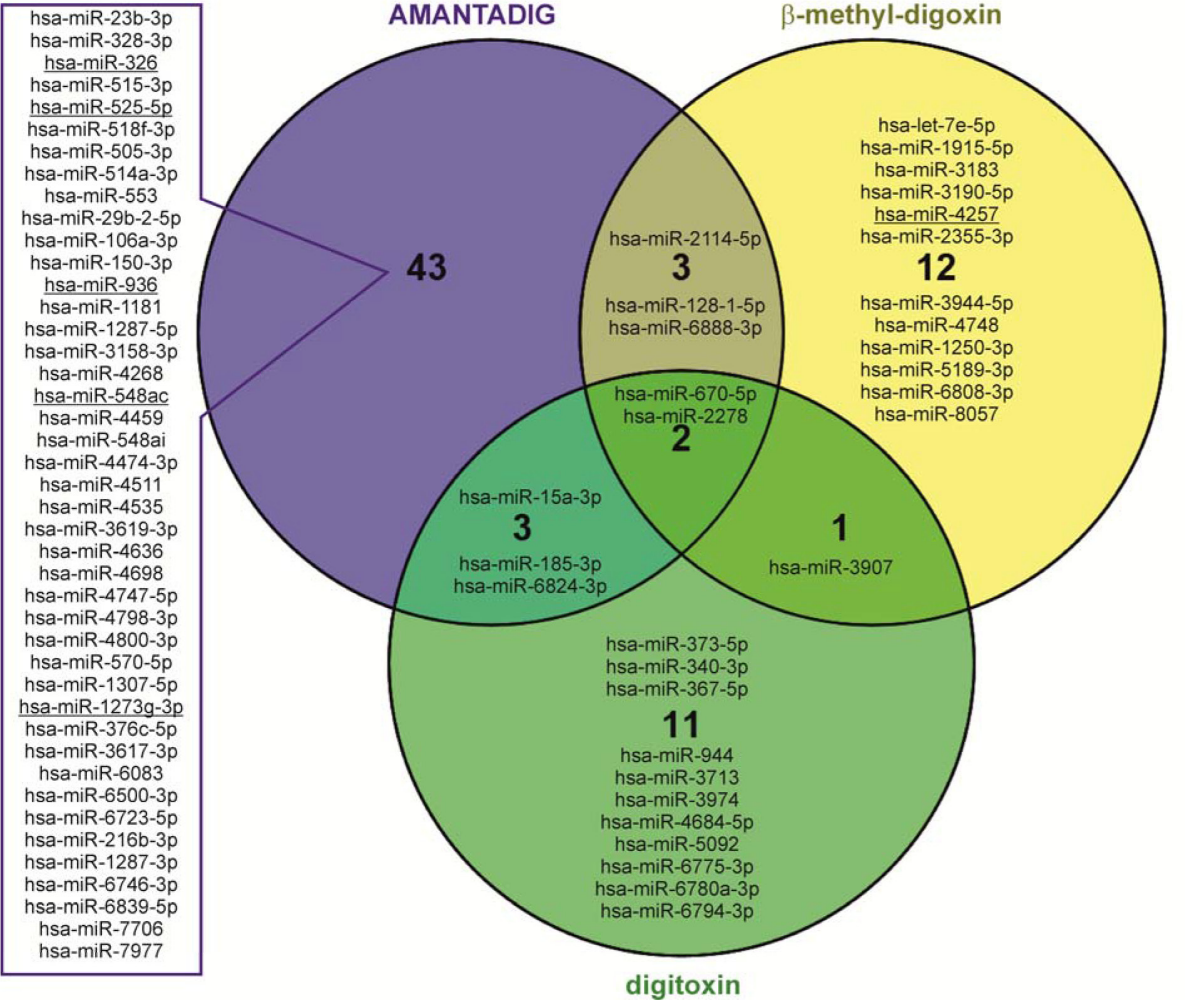
Venn diagram showing overlapping miRNAs Venn diagram showing overlapping mature miRNAs after treatment with all three cardiac glycosides in all four RCC cell lines. Underlined miRNAs are predicted in silico to target survivin.

To better understand the effects of cardiac glycosides, including the molecular basis of G2/M arrest and involved signaling pathways, we performed miRNA microarray analyses. We applied IC50 concentrations of AMANTADIG, digitoxin and ß-methyl-digoxin to all four cell lines for 72 h. We detected a distinct clustering of the miRNA expression profiles according to the applied treatment, showing that each cardiac glycoside was able to induce characteristic changes in the miRNA expression patterns (Supplementary Table 1). Compared with untreated controls, digitoxin treatment resulted in 7 up- and 10 downregulated miRNAs, ß-methyl-digoxin treatment resulted in 3 up- and 15 downregulated miRNAs, and AMANTADIG treatment resulted in 17 up- and 34 downregulated miRNAs (Figure 3 and Supplementary Figure 2). Two miRNAs showed overlap between all treatments and all cell lines: miR-670-5p and miR-2278 (Figure 4).

### miR-2278 and miR-670-5p expression

**Figure 5:**
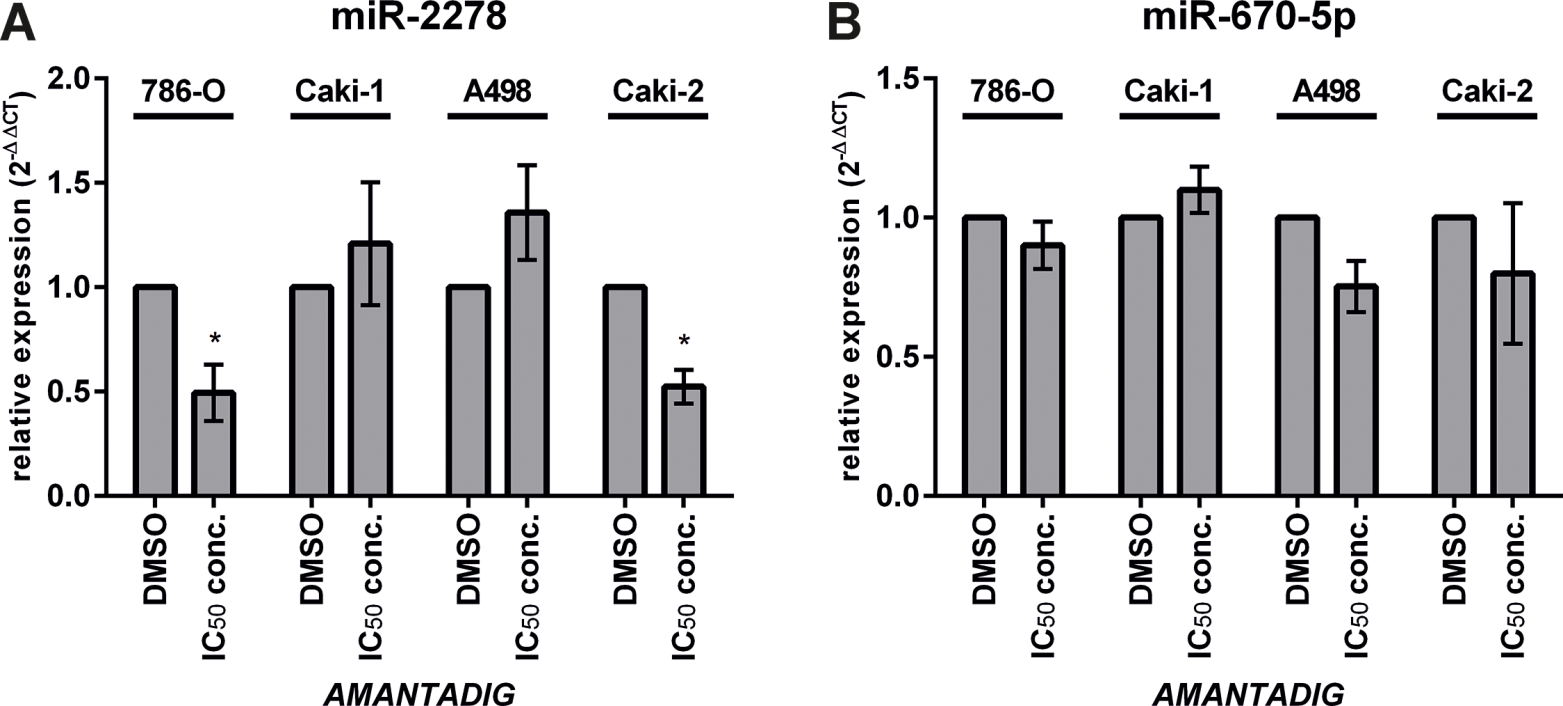
Expression of miR-2278 and miR-670-5p The expression of **(A)** miR-2278 and **(B)** miR-670-5p in four RCC cell lines was measured using quantitative real-time PCR after treatment with AMANTADIG. Asterisks indicate p < 0.05.

Treatment with AMANTADIG at the IC50 concentration significantly decreased the expression of miR-2278 in the cell lines 786-O and Caki-2, but not in Caki-1 and A498 cells, compared with the untreated control cells (Figure 5A). Although expression of miR-670-5p was not significantly reduced in any cell line treated with AMANTADIG, slight decreases were detected in 786-O, A498 and Caki-2 cells (Figure 5B).

### *In silico* prediction of miRNA target genes

Five programs were used to predict the target genes of all significantly deregulated miRNAs *in silico*: miRWalk, Microt4, miRanda, RNAhybrid, and Targetscan. A gene was considered a potential target of a significantly deregulated miRNA only when all five prediction programs identified it concordantly. However, the *in silico* analysis revealed 2771 potential miRNA target genes. To further elucidate the pathways that contain these genes, we applied pathway enrichment analysis (Supplementary Table 1).

### Pathway enrichment analysis

We performed pathway enrichment analysis using three different programs: WIKI, KEGG and REACTOME. We considered only pathways that were predicted to be significantly affected. We identified 7, 2 and 3 pathways to be significantly affected by all three treatments in all four cell lines using WIKI, KEGG and REACTOME, respectively (Supplementary Table 1). Interestingly, the KEGG program identified several cancer-associated genes/pathways in AMANTADIG-treated cells (pathways in colorectal, pancreatic cancer, glioma, melanoma and chronic myeloid leukemia; Supplementary Table 1). A comparison of the three programs showed that two programs consistently produced overlapping results for the MAPK pathway (WIKI and KEGG) and the axon guidance pathway (KEGG and REACTOME). The programs WIKI and REACTOME showed no overlaps in pathway predictions.

**Figure 6:**
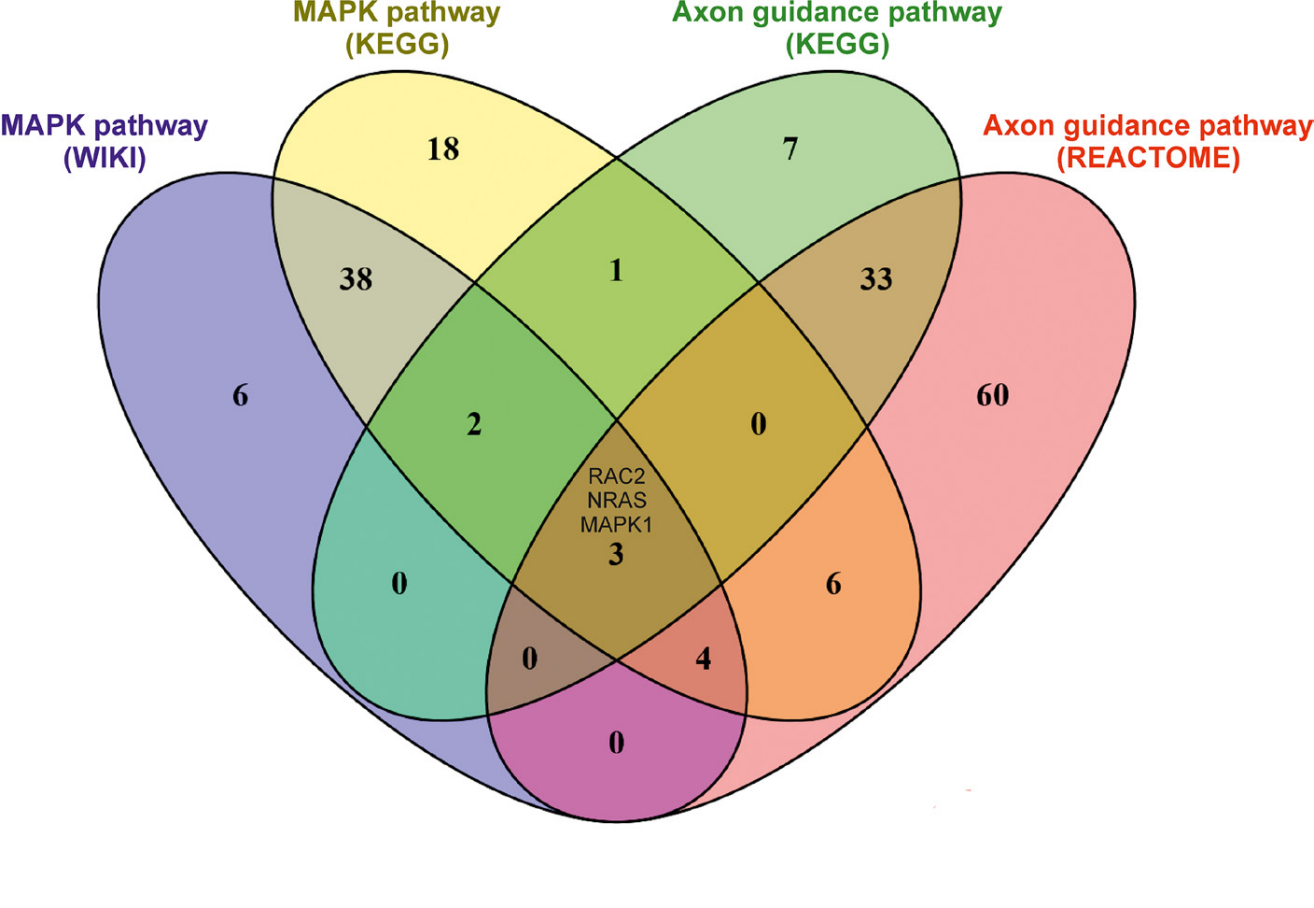
Venn diagram showing the overlap of genes identified for the MAPK pathway and the Axon guidance pathway A pathway enrichment analysis of in silico target genes of deregulated miRNAs was performed using WIKI, KEGG and REACTOME. The Venn diagram shows overlapping genes of the MAPK pathway (WIKI, KEGG) and the axon guidance pathway (KEGG, REACTOME). MAPK, NRAS and RAC2 were detected by the pathway enrichment analyses of WIKI and KEGG for the MAPK signaling pathway and by the pathway enrichment analyses of KEGG and REACTOME for the axon guidance pathway.

Next, we searched for overlaps between the identified signaling pathways in terms of the genes predicted to be regulated by miRNAs and for genes that overlapped between the different prediction programs (Supplementary Table 1). Interestingly, three prominent genes belonging to the MAPK pathway and the axon guidance pathway were targets of miRNAs deregulated in all cell lines under all treatment conditions. These genes were MAPK1/ERK2, NRAS and RAC2 (Figure 6). MAPK1 and NRAS are putative target genes of miR-2278 (Table 2). AMANTADIG, digitoxin and ß-methyl-digoxin treatments significantly downregulated miR-2278 expression compared with that of untreated control cells (DMSO) by 0.566-fold, 0.647-fold and 0.551-fold, respectively (Table 2). RAC2 is predicted to be downregulated by miR-670-5p. Accordingly, AMANTADIG, digitoxin and ß-methyl-digoxin treatment significantly downregulated the expression of this gene by 0.464-fold, 0.371-fold and 0.485-fold, respectively (Supplementary Table 1). Both MAPK1 and NRAS have been reported to play a role in G2 cell cycle checkpoint function [24, 25]. RAC2, a member of the RAS superfamily of small GTP-binding proteins, appears to stimulate cell growth, cytoskeletal reorganization, and the activation of protein kinases, and a connection to the MAPK/ERK pathway has been described [26].

**Table 2:** Deregulated miRNAs after cardiac glycoside treatment targeting MAPK1 in silico

Comparison	miRNA	ANOVA adjusted (*p*-value)	fold change	fold change (log2)
A vs. C	hsa-miR-548ac^1, 2^	0.045	1.792	0.841
A vs. C	hsa-miR-936^1^	0.034	1.766	0.821
A vs. C	hsa-miR-4511	0.037	1.735	0.795
A vs. C	hsa-miR-518f-3p	0.030	1.638	0.712
A vs. C	hsa-miR-1273g-3p^1^	0.021	1.142	0.192
A vs. C	hsa-miR-23b-3p	0.025	0.982	–0.026
A vs. C	hsa-miR-525-5p^1, 2^	0.029	0.650	–0.621
A vs. C	hsa-miR-150-3p	0.043	0.639	–0.645
A vs. C	hsa-miR-1287-3p	0.046	0.575	–0.799
A vs. C	hsa-miR-2278^2^	0.015	0.566	–0.821
A vs. C	hsa-miR-4698	0.029	0.506	–0.982
A vs. C	hsa-miR-29b-2-5p	0.002	0.468	–1.096
A vs. C	hsa-miR-185-3p	0.020	0.379	–1.398
A vs. C	hsa-miR-326^1^	0.023	0.369	–1.437
D vs. C	hsa-miR-944^2^	0.049	1.469	0.556
D vs. C	hsa-miR-2278^2^	0.049	0.647	–0.629
D vs. C	hsa-miR-185-3p	0.036	0.440	–1.184
M vs. C	hsa-miR-3183	0.049	0.658	–0.603
M vs. C	hsa-miR-2278^2^	0.012	0.551	–0.861
M vs. C	hsa-miR-4257^1^	0.034	0.509	–0.974

Abbreviations: A = AMANTADIG, C = Control, D = digitoxin, M = ß-methyl-digoxin

All miRNAs target *in silico* MAPK (but hsa-miR-936) at including miRNAs that target *in silico*^1^survivin, ^2^NRAS.

### MAPK1 mRNA and protein expression

**Figure 7:**
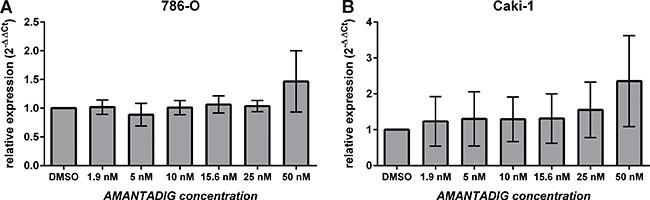
Expression of MAPK1 mRNA **(A)** 786-O and **(B)** Caki-1 cells were treated with the indicated concentrations of AMANTADIG for 48 h. MAPK1 expression was determined by quantitative real-time PCR. The values represent the mean and standard deviation of four independent experiments. We applied One-way Anova and Tukey multiple comparison test corrected for multiple testing.

**Figure 8:**
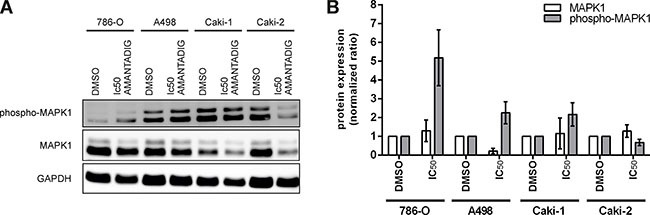
Protein expression of MAPK1 786-O, Caki-1, A498, and Caki-2 cells were treated with the IC50 value of AMANTADIG for 48 h. **(A)** Representative western blotting images for phosho-MAPK1, MAPK1 and the loading control GAPDH. **(B)** Quantification of four independent experiments.

The next RNA/protein studies, we focused on Caki-1 and 786-O cells since Caki-1 cells were the most sensitive cells and 786-O together with Caki-2 cells were comparably on the second position in their sensitivity towards AMANTADIG treatment (Figure 1). Since 786-O cells are more often applied as RCC model than Caki-2 cells we decided to study them in addition to Caki-1 cells. MAPK1 mRNA remained unchanged, independent of the concentration of AMANTADIG applied to both 786-O and Caki-1 cells (Figure 7). Comparably, the total level of MAPK1 protein did not change in response to any of the concentrations of AMANTADIG applied to 786-O and Caki-1 cells. However, the expression of phosphorylated MAPK1 (pMAPK1) significantly increased in both cell lines in response to 15 nM and 25 nM AMANTADIG compared to that of control cells. A further increase in the abundance of pMAPK1 in response to 50 nM AMANTADIG was observed in 786-O cells but not in Caki-1 cells (Figure 8).

### miRNAs targeting survivin

To supplement our results, we assessed the effect of miRNAs that are deregulated in response to treatment with cardiac glycosides on a gene known to be involved in G2/M phase. Thus, we selected survivin (BIRC5), whose expression has been described to be altered after digitoxin treatment [7]. Moreover, this gene has also been described as a G2/M cell phase regulator [27]. Again, we utilized the five target prediction programs, and the respective miRNAs were considered only when all programs identified *survivin* as a miRNA target gene. After AMANTADIG treatment, five miRNAs were found to be able to target *survivin*: hsa-miR-548ac, hsa-miR-1273g-3p and hsa-miR-936, which were upregulated; and hsa-miR-326 and hsa-miR-525-5p, which were downregulated. Furthermore, after treatment with ß-methyl-digoxin, one candidate miRNA was found to be downregulated: hsa-miR-4257 (Table 2). Interestingly, 5 miRNAs (miR-326, miR-525-5p, miR-548ac, miR-1273g-3p and miR-4257) are predicted to downregulate both *survivin* and *MAPK1*, and 2 miRNAs (miR-525-5p and miR-548ac) are predicted to downregulate both *survivin* and *NRAS* (Table 2), supporting the hypothesis that these deregulated miRNAs are involved in G2/M cell cycle arrest, possibly in a concerted manner.

### *Survivin* mRNA expression after treatment with AMANTADIG

**Figure 9:**
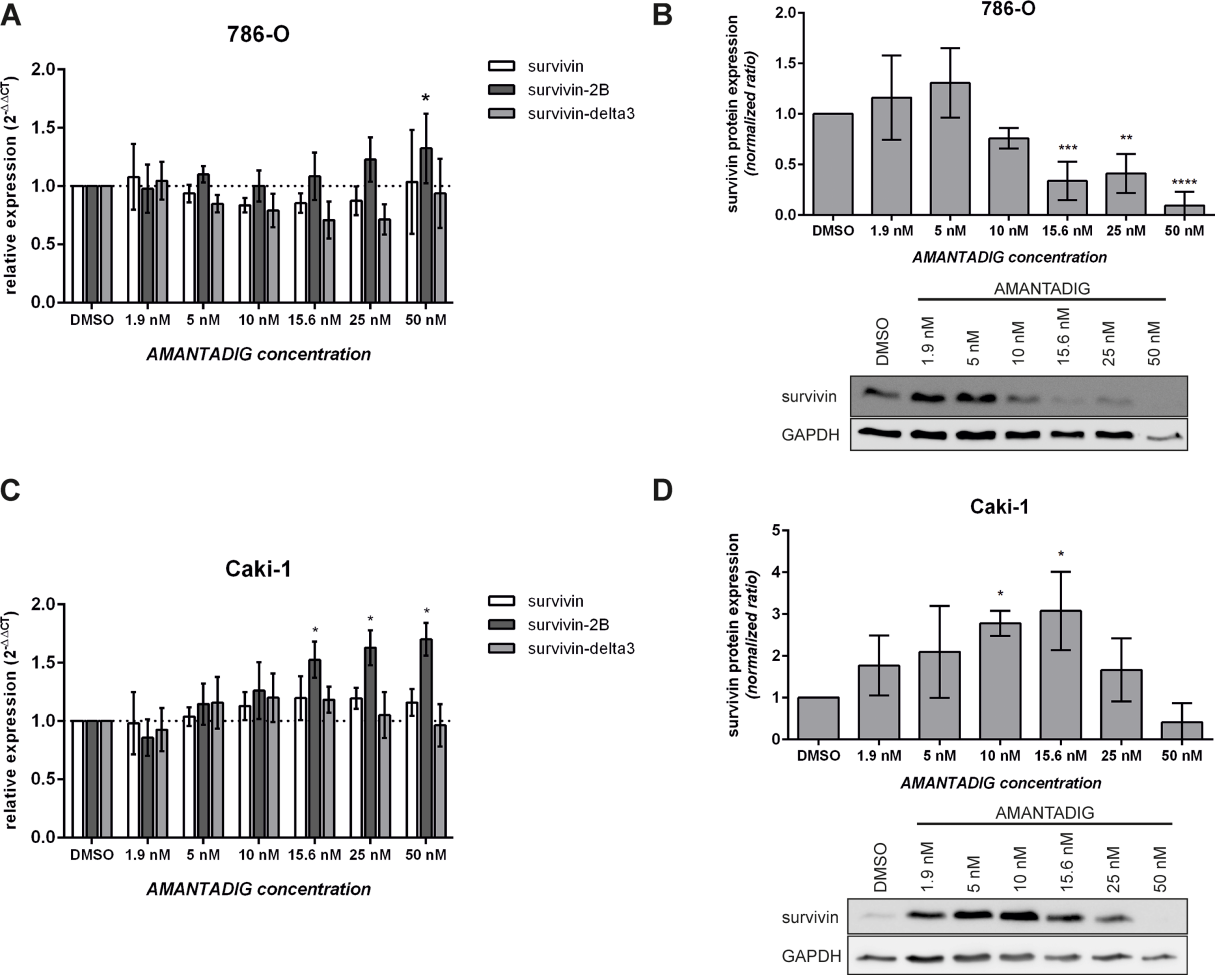
Expression of survivin splice variants and survivin protein 786-O and Caki-1 cells were treated with the indicated concentrations of AMANTADIG for 48 h. **(A, C)** Survivin expression was determined by quantitative real-time PCR. The values represent the mean and standard deviation of four independent experiments. **(B, D)** Representative western blotting images and quantification of four independent experiments showing survivin protein expression. Asterisks indicate p < 0.05.

*Survivin* is expressed as the wild type and various splice variants [reviewed in 28]. Whereas wild type *survivin* and *survivin-delta3* are considered anti-apoptotic, *survivin-2B* is considered pro-apoptotic [29]. To delineate the expression of *survivin* variants, 786-O and Caki-1 cells were treated with different concentrations of AMANTADIG and compared to control cells. After incubating the cells for 48 h with AMANTADIG, we studied the mRNA expression of wild-type *survivin*, *survivin-2B* and *survivin-delta3*. We found that treatment with 50 nM and 15.6 nM AMANTADIG significantly increased the expression of *survivin-2B* in 786-O cells and Caki-1 cells, respectively (Figure 9A and 9C). However, the mRNA levels of wild-type *survivin* and *survivin-delta3* were not significantly changed after AMANTADIG treatment in both these renal cell carcinoma cell lines.

### Survivin protein expression after treatment with AMANTADIG

Wild-type survivin is predominantly expressed at the mRNA level, unlike the survivin-delta3 and survivin-delta-2B isoforms [30], which suggests that the majority of survivin protein is wild-type survivin. We detected only one dominant band at approximately 17 kDa, which is thought to represent wild-type survivin. Treatment with AMANTADIG concentrations of 15.6 nM, 25 nM and 50 nM resulted in significantly lower survivin expression in 786-O cells compared to control cells (Figure 9B). The expression of survivin protein in Caki-1 cells was somewhat ambiguous. Cells treated with AMANTADIG concentrations of 10 nM or 15.6 nM showed significantly increased survivin expression, whereas treatment with 50 nM AMANTADIG resulted in a strong but not significant reduction of survivin protein expression (Figure 9D).

## DISCUSSION

Cardiac glycosides have been shown to inhibit cancer cell growth mainly by inhibiting the Na^+^/K^+^-ATPase signalosome which controls various cellular-physiological activities, such as apoptosis, cell proliferation, autophagy, radical oxygen species production, cell motility and cell cycle arrest [reviewed in 5, 31]. Accordingly, we could show that increasing concentration of cardiac glycosides was associated with a decrease of activity of Na^+^K^+^- ATPase and an increase of cytotoxicity in RCC cell lines. (Supplementary Figure 1; Figure 1).

In this study, we analyzed the effect of treatment with three cardiac glycosides, digitoxin, ß-methyl-digoxin and AMANTADIG, on four renal cell lines in terms of (i) cell cycle arrest, (ii) miRNA profiles and the consequent (iii) expression of genes involved in cell cycle regulation.

The G2/M cell cycle phase is of special therapeutic interest because different tumor cells respond most effectively to radio- and/or chemotherapy during this phase [32–35]. Cardiac glycosides have been previously described to inhibit the cell cycle; specifically, to arrest cells in the G2/M phase [6, 7, 36–38]. Accordingly, G2/M arrest was observed in Caki-2 cells after digitoxin treatment and in 786-O, Caki-1 and Caki-2 cells after ß-methyl-digoxin treatment. In addition, we showed for the first time that AMANTADIG can induce G2/M arrest in renal cell carcinoma cell lines. However, the molecular basis underlying this cardiac glycoside-induced cell cycle arrest remains unclear.

This study consisted of two approaches. First, we analyzed the microRNA expression profiles of four renal cell carcinoma cell lines after treating them with AMANTADIG and the other two cardiac glycosides. Second, we studied the regulation of survivin in these tumor cells after cardiac glycoside treatment because some cardiac glycosides are known to induce G2/M arrest via survivin [7, 39].

After analyzing the miRNA microarray data, we showed that miR-670-5p and miR-2278 are significantly downregulated for all cardiac glycoside treatments in the RCC cell lines. To date, experimentally validated target genes for both miRNAs have not yet been reported.

To further elaborate the role of deregulated miRNAs caused by cardiac glycoside treatments, we first identified in silico targets of the miRNAs, and then these putative miRNA targets were used as inputs for pathway enrichment analysis. Our analyses suggested that the deregulated miRNAs can target MAPK pathway through regulating the expression of *MAPK1*, *NRAS* and *RAC2*.

To verify the computational result, we investigated the mRNA and protein level of MAPK1, an *in silico* target of miR-2278, in 786-O and Caki-1 cell lines treated with AMANTADIG. MAPK1 acts as an oncogene by promoting cell survival, cell proliferation and cell motility but it also plays a crucial role in cell cycle arrest when DNA damage is triggered [24, 25, 40–42]. Our data showed the unchanged expression levels of MAPK1 mRNA and protein but significant increase of phosphorylated MAPK1 (pMAPK1), which is the functionally active form of MAPK1. This result is in agreement with previous publications that cardiac glycoside (ouabain) does not change total MAPK1 (ERK2) but increases pMAPK1 protein expression in rat renal epithelial cells, neuroblastoma cells and lung cancer cells [43–45].

In our second approach, we studied the mRNA and protein expression of survivin. A G2/M-associated downregulation of survivin has been described for the cardiac glycosides calotropin, digitoxin and D6-MA [7, 39]. Survivin (BIRC5) is a known apoptosis inhibitor and promotes cell proliferation [46, 47]. Moreover, survivin, whose expression is predominant in the G2/M cell phase, supports passage through the pro-meta and metaphase of cell cycle and ensures cell division/proliferation [47, 48]. Interestingly, survivin is expressed in many human cancer tissues but to a much lesser extent in normal differentiated tissues. Thus, survivin is a promising target in cancer therapy [49–51]. We showed that cardiac glycosides may be used to effectively inhibit survivin and its anti-apoptotic splice variant(s), what is consistent with previous studies [4, 7]. Treatment with AMANTADIG significantly reduced the survivin protein levels in 786-O cells (p53- and Vhl-mutated) but not in Caki-1 cells (p53- and Vhl-wildtype). Taken together, our results suggest that the effect of AMANTADIG on survivin expression can be cell line specific.

Recently, survivin splice variants and their cell biological functions have been reviewed [28]. Whereas survivin and survivin-delta3 are anti-apoptotic, survivin-2B has lost its anti-apoptotic potential and may act as a naturally occurring antagonist of survivin and survivin-delta3 [29, 30]. The survivin variants survivin-delta3 and survivin-2B can heterodimerize with survivin and consequently regulate the balance between proliferation and cell death [52]. Remarkably, we observed a significant increase in survivin-2B mRNA after treating 786-O and Caki-1 cell lines with AMANTADIG. This work is the first to describe an increase in survivin-2B mRNA expression after cardiac glycoside treatment. Moreover, the expression level of survivin-2B/survivin in stage III and IV colorectal cancer was lower than that in stage I and II tumors [53], and reduced survivin-2B mRNA expression has been associated with colorectal cancer in the advanced pT stages [54]. In addition, a higher level of survivin-2B/survivin significantly correlated with a better prognosis [53]. Lower survivin-2B levels were also associated with advanced tumor grades and stages in bladder cancer [55]. For RCC, data on *survivin-2B* mRNA are somewhat controversial, whereas a decrease in the ratio of *survivin-2B* to *survivin* mRNA was observed in Caucasian RCC patients in late tumor stages. In an Asian RCC cohort, the *survivin-2B* gene expression levels were significantly higher in pT3 RCC than in pT1 tumors [56]. In addition, the survivin-2B variant has been shown to be activated by p53 and to sensitize acute lymphocytic leukemia cells to chemotherapy with doxorubicin [57]. However, in acute myeloid leukemia patients, higher *survivin-2B* mRNA expression levels were associated with a refractory response to chemotherapy [58]. Overall, the expression of survivin-2B depends on ethnicity and tumor type and may consequently have different implications for tumor treatment with chemotherapy. However, the protein expression of survivin-2B could not be studied because an efficient survivin-2B antibody is not available.

Tumor cells can most effectively respond to radio- and/or chemotherapy during the G2/M-phase [32–35]. Cardiac glycosides can arrest RCC cells in the G2/M cell phase, and thus speculating that treatment with cardiac glycosides can sensitize RCC cells to radio and/or chemotherapy is tempting. Furthermore, combination treatment with cardiac glycosides and radio-/chemotherapies is of great interest for cancer patients but certainly requires further studies in the future.

In summary, the treatment of RCC cells with cardiac glycosides, especially AMANTADIG, inhibited cell proliferation by increasing the number of cells in the G2/M cell cycle phase. Moreover, miRNA microarray analyses revealed that miR-670-5p and miR-2278 were downregulated in RCC cells in response to treatment with various cardiac glycosides. In addition, we identified the MAPK signaling pathway as a common target of these miRNAs in RCC cell lines. Specifically, AMANTADIG treatment significantly upregulated pMAPK1 expression in 786-O cells. Furthermore, AMANTADIG treatment significantly increased the expression of the pro-apoptotic splice variant survivin-2B in RCC cell lines. These data suggest a regulatory network between the identified miRNAs and their target genes/proteins that affects cell cycle regulation. Moreover, our results may provide a basis to study combined treatment consisting of cardiac glycosides and radio-/chemotherapy for RCC in the future.

## MATERIALS AND METHODS

### Materials

All chemicals were of reagent grade. Digitoxin, β-methyl-digoxin and AMANTADIG were obtained from the substance collection available from the Chair of Pharmaceutical Biology, University of Erlangen-Nuremberg. The new semisynthetic cardenolide analog AMANTADIG ((3β-[2-(1-amantadine)-1-on-ethylamine]-digitoxigenin) was previously described [11]. The compounds were dissolved in DMSO at concentrations of 20 mM and stored at 4°C. All other chemicals were obtained from Carl Roth (Karlsruhe, Germany).

### Cell lines

The renal cell carcinoma cell lines (A498, 786-O, Caki-1, Caki-2) were obtained from the German collection of microorganisms and cell cultures (DSMZ, Braunschweig, Germany). The cells were cultured in DMEM medium (Sigma-Aldrich, Munich, Germany) supplemented with 10% heat-inactivated fetal bovine serum, 2 mM/L L-glutamine, 1 g/L glucose, 100 U/mL penicillin, and 100 μg/mL streptomycin at 37°C in a humidified atmosphere of 5% CO_2_.

### Cell viability

The colorimetric MTT assay was performed using 3-(4,5-dimethyl-1,3-thiazole-2-yl)-2,5-diphenyltetrazolium bromide (MTT). Cells (6000 renal cell carcinoma cells per well of a 96-well microtiter plate) were cultured for 24, 48 and 72 h with various concentrations of the compounds. At the indicated time points, MTT was added to a final concentration of 1 mg/ml, and 200 μl of DMSO was added after 4-h incubation at 37°C to dissolve the formazan crystals. The absorbance was measured at 570 nm using a plate spectrophotometer (VersaMax ELISA Microplate Reader, Molecular Devices, USA). Cells treated with 0.5% DMSO served as a negative control to define 100% viability. The percentage of viable cells was plotted against the drug concentration, and the IC50 values were determined based on the dose-response curves using GraphPad Prism 6.0 (Graph Pad software, La Jolla, CA).

### Cell cycle analysis

To assess cell cycle distribution, cells (9 × 10^4^) were seeded in 6-well plates and with either digitoxin, β-methyl-digoxin or AMANTADIG at concentrations corresponding to the IC50 value. The cells were harvested after 48 h of incubation and fixed in 90% Methanol/10% PBS at –20°C. After fixation, the cells were treated with 100 μg/ml RNAse A and stained with 50 μg/ml propidium iodide (PI) at 4°C overnight. The cells were then analyzed using a FACS Canto II instrument (Becton Dickinson, BD, USA), and the percentages of cells in each phase of the cell cycle (G1, S, and G2/M) were determined using the FlowJo software v7 (FlowJo, LLC, Ashland, OR, USA).

### RNA and protein extraction

Total RNA was isolated using TRIzol (Invitrogen, Darmstadt, Germany) according to the manufacturer's instructions. To extract protein, the cells were lysed with RIPA lysis buffer (25 mM Tris-HCl pH 8.0, 137 mM NaCl, 10% glycerol, 0.1% SDS, 0.5% sodium deoxycholate, 1% NP40, 2 mM EDTA pH 8.0, 1 mM sodium vanadate, and 1.5 mM sodium fluoride) for 15 min, and the cell lysates were clarified by centrifugation.

### miRNA microarrays

miRNA expression was measured on GeneChip miRNA microarrays V4.0 (Affymetrix, Santa Clara, CA, USA) according to the manufacturer's instructions. The array contained sequence-specific probes for 2,578 human miRNAs listed in miRBase v20.0 (http://www.mirbase.org). The signal intensity data were further analyzed with the Partek software v6.2 (Partek, St Louis, MO, USA).

### Quantitative real-time PCR (qRT-PCR) analysis of miRNA expression

For the miRNA analysis, total RNA was reverse transcribed using the miScript II RT Kit (Qiagen, Hilden, Germany). Real-time PCR was carried out using the miScript SYBR Green PCR Kit and QuantiTect Primer Assays for miR-2278, miR-670-5p, miR-28-5p and miR-103a-3p (Qiagen). Real-time PCRs were performed in triplicate in a final volume of 10 μl with the StepOnePlus Real-Time PCR System (Life Technologies, Darmstadt, Germany). The relative RNA expression levels were calculated by applying the ΔΔCt method using miR-28-5p and miR-103a-3p as reference miRNAs [59].

### qRT-PCR analysis of mRNA expression

First-strand cDNA was synthesized using the DyNAmo cDNA Synthesis Kit (Thermo Fisher Scientific, Waltham, USA). The mRNA transcripts were detected using PrimeTime qPCR assays (Integrated DNA Technologies, IDT, Leuven, Belgium). Sequence-specific primers and fluorescence-labeled probes complementary to a sequence present in all *survivin* splice variants (Hs.PT.56a.1608989.g), *survivin-2B* (Hs.PT.56a.3536061), *survivin-Δex3* (Hs.PT.56a.21530439), *MAPK1* (Hs.PT.58.39782850), and the endogenous controls *GAPDH* (Hs.PT.39a.22214836) and *HPRT1* (Hs.PT.58.20881146) were used. Real-time PCRs were performed in triplicate in a final volume of 10 μl containing 1× TaqMan Fast Universal master mix (Life Technologies) and 1xPrimeTime assay. The relative RNA expression levels were calculated by applying the ΔΔCt method [59].

### Western blotting analysis

Equal amounts of total protein lysates were separated by SDS-PAGE and transferred to nitrocellulose membranes (GE Healthcare, Freiburg, Germany) by electroblotting. Primary monoclonal antibodies against p44/42 MAPK, phospho-p44/42 MAPK (Thr202/Tyr204), and GAPDH were purchased from Cell Signaling Technology (Danvers, USA). Polyclonal anti-survivin antibody was purchased from R&D systems (Abingdon, UK). Secondary anti-rabbit and anti-mouse antibodies conjugated with horseradish peroxidase were purchased from Jackson ImmunoResearch (Suffolk, UK). Protein bands were revealed by Western BLoT Ultra Sensitive HRP Substrate, Clontech, Saint-Germain-en-Laye, FR) in an LAS-4000 chemiluminescence detection system (Raytek, Sheffield, UK).

### Na^+^/K^+^-ATPase assay

Enzymatic activities of Na^+^/K^+^-ATPase α1,2,3 subunit of porcine cortex (Sigma) were assayed using 4 mM ATP as substrate in a final volume of 40 μL assay puffer containing 40 mM Tris-HCl pH 7.5, 80 mM NaCl, 1 mM EDTA, 8 mM MgAc_2_. Negative control was assayed without enzyme and 4 mM ATP was added after 30 min of incubation at room temperature. Positive control contained 0.05 U/mL of Na^+^/K^+^-ATPase α1, 2, 3 subunit of porcine cortex in a 30 min pre-incubated mixture with assay puffer after 4 mM ATP was added. Inhibition assays were performed by co-incubating enzyme and inhibitor with increasing concentrations from 0.2 μM–1000 μM for 30 min and adding of 4 mM ATP solution. Reactions were stopped after 30 min of incubation and activity was determined by measuring the Pi released according to the malachite-green test [60]. Activity was scored as the percentage of reduction of absorbance subtracting the absorbance at 600 nm of the control well, relative to the positive control well. Positive control defined 100% enzyme activity. All experiments were performed in triplicates and the results were expressed at the mean of IC50 values (drug concentration that reduced enzyme activity to 50%).

### Statistical analyses

All data of the Na^+^/K^+^-ATPase Assay are expressed as mean ± standard deviation of the mean. Means between the various groups were compared by two-way ANOVA analysis. In case of multiple comparisons, a post hoc Bonferroni correction was applied. *P value*s < 0.001 were considered statistically significant. Data were analyzed using GraphPad Prism 5 Software (GraphPad, San Diego, CA, USA)

### Microarray data processing and analyses

The following methods were used to identify miRNAs that are deregulated in renal cancer cell lines in response to different cardiac glycosides. The function *celintensityread* in the Matlab bioinformatics toolbox (version 2014b) was applied to read *.cel files generated by the Affymetrix miRNA 4.0 array. In addition, the function *affysnpannotread* was modified to read the annotation file for the array. As a result, a probe intensity matrix of 337504 rows and 16 columns was obtained. The rows and columns of the matrix represent the probe sets and individual samples designed on the array, respectively. Next, the function *affysnpannotread*, which uses the robust multi-array average method, was applied to process the probe intensity matrix. This method first subjected the intensity matrix to background adjustment, quantile normalization and log2 transformation, resulting in an expression matrix of 36249 rows and 16 columns. The rows and columns of the matrix represent features (e.g., *Homo sapiens* miRNAs) and individual samples, respectively. Parts of the expression matrix corresponding to the expressions of *Homo sapiens* mature miRNAs were extracted, and a one-way ANOVA (*anova1*) was performed to identify deregulated miRNAs for different comparisons. The *p*-values for multiple comparisons (*multcompare*) were corrected using Tukey's honestly significant difference procedure with a significance value ≤ 0.05. The miRNAs with corrected *p*-values ≤ 0.05 were selected as significantly deregulated miRNAs.

### miRNA target genes

The miRwalk2.0 database was used to identify the target genes of the identified deregulated miRNAs [61]. The miRNA target genes predicted *in silico* by at least five independent algorithms, including miRwalk2.0, DIANA-microTv4.0, MiRanda (release 2010), HybridRNA (version 2.1) and Targetscan (version 6.2), were extracted. These algorithms predict miRNA target genes based on common features, such as seed region match, conservation, free energy, and site accessibility on the 3’ UTRs of target gene mRNAs by mature miRNA sequences [62, 63]. This strategy ensures more confidence in putative genes targeted by the deregulated miRNAs than results from a single miRNA target prediction algorithm, which usually contains many false positive miRNA targets [64].

### Pathway enrichment analyses

The obtained miRNA target genes were further used as inputs for pathway enrichment analyses using the tool Enrichr [65]. The tool provides significant biological pathways associated with the given miRNA target genes using Fisher's exact test, which assumes a binomial distribution and independence for the probability of any gene belonging to any set. The results are lists of curated pathways to which given miRNA target genes belong from well-established databases, such as KEGG [66], WikiPathways [67] or Reactome [68]. The pathways with corrected *p*-values ≤ 0.05 were selected as significant.

## SUPPLEMENTARY MATERIALS FIGURES AND TABLES




