# Supplementary material for: A new semisynthetic cardenolide analog 3β-[2-(1-amantadine)- 1-on-ethylamine]-digitoxigenin (AMANTADIG) affects G2/M cell cycle arrest and miRNA expression profiles and enhances proapoptotic survivin-2B expression in renal cell carcinoma cell lines
Source: Oncotarget. 2017 Jan 14;8(7):11676–91. doi: 10.18632/oncotarget.14644 (PMC5355295; doi:10.18632/oncotarget.14644)
Supplement: Supplementary file 1 [file oncotarget-08-11676-s001.pdf]

A new semisynthetic cardenolide analog 3β-[2-(1-amantadine)-1-on-ethylamine]-digitoxigenin (AMANTADIG) affects G2/M cell cycle arrest and miRNA expression profiles and enhances pro-apoptotic survivin-2B expression in renal cell carcinoma cell lines

Supplementary Materials

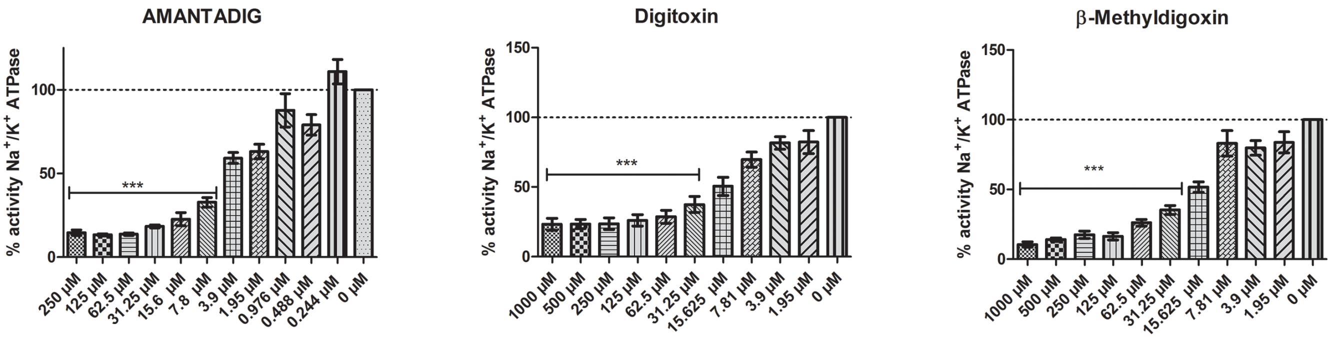

IC50 [µM]	95 % CI
4.780	3.773 to 6.056

P<0.001

IC50	95 % CI
12.14	9.043 to 16.29

P<0.001

IC50	95 % CI
16.73	13.70 to 20.43

P<0.001

Supplementary Figure 1: Inhibition of Na^+/K^+-ATPase activity by cardiac glycosides. Effects of AMANTDIG, digitoxin and β-methyldigoxin on Na^+/K^+-ATPase activity were assayed with Na^+/K^+-ATPase α1,2,3 subunit of porcine cortex. Results indicated an increasing affinity towards Na^+/K^+-ATPase inhibition from β-methyldigoxin, digitoxin to AMANTADIG, with IC50 values ranking between 16.73 µM and 4.78 µM.

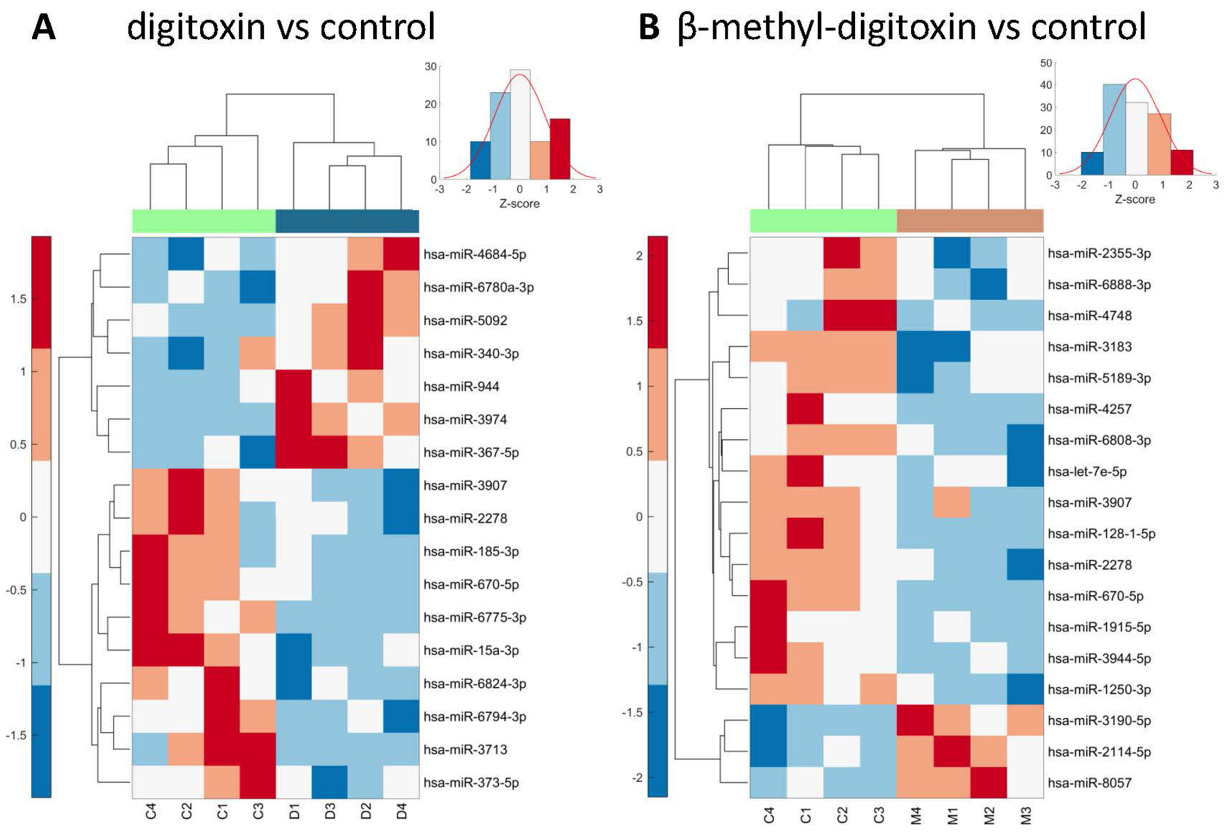

Supplementary Figure 2: Heat map of deregulated miRNAs for digitoxin and ß-methyl-digoxin treatment. Heat map of deregulated miRNAs after treating the four RCC cell lines with DMSO [C], digitoxin [D] or ß-methyl-digoxin [M]. 1: A498; 2: 786-O; 3: Caki-1; and 4: Caki-2

Supplementary Table 1: Bioinformatic analysis results. See Supplementary_Table 1
